# Supplementary material for: A distributed nanocluster based multi-agent evolutionary network
Source: Nat Commun. 2022 Aug 10;13:4698. doi: 10.1038/s41467-022-32497-5 (PMC9365837; doi:10.1038/s41467-022-32497-5)
Supplement: Supplementary file 1 — Supplementary Information [file 41467_2022_32497_MOESM1_ESM.pdf]

Supplementary Information

A Distributed Nanocluster Based Multi-Agent Evolutionary Network

Xu et al.

Fabrication of Ag nanocluster based multi-agent evolutionary network system

The device fabrication started from silicon wafers with 300 nm thermally grown silicon dioxide. Water molecules absorbed on SiO_2 surface were expelled by heating wafer at 170 °C for 10 min. Afterwards, PMMA as the positive resist was covered on the wafer surface using spin coating (3000 r/min, 1 min), followed by a series of processes consisting of electron-beam lithography, development (IPA/MIBK with a volume ratio of 3:1), electron beam evaporation and lift-off to form the pattern of metal electrodes. The metal materials Ti (1 nm)/Au (40nm) were used for the fabrication of metal electrodes in this study. To get the polymer electrolyte, polyethylene oxide (PEO, 100,000 g/mol) was dissolved in acetonitrile by 5wt.‰. The homogeneous solution was then dropped above the surface of device and the solvent was removed using high-speed spin coating to form the solid polymer electrolyte with self-assembled lamellar structures, providing long-range Ag^+ ion transport paths (Supplementary Ref. S1). Subsequently, discrete Ag nanoclusters were incorporated into the solid polymer electrolyte by electron beam evaporation with a thickness of 3 nm below filming condition. The fabrication process of device with two terminals is schematically shown in Supplementary Fig. 1.

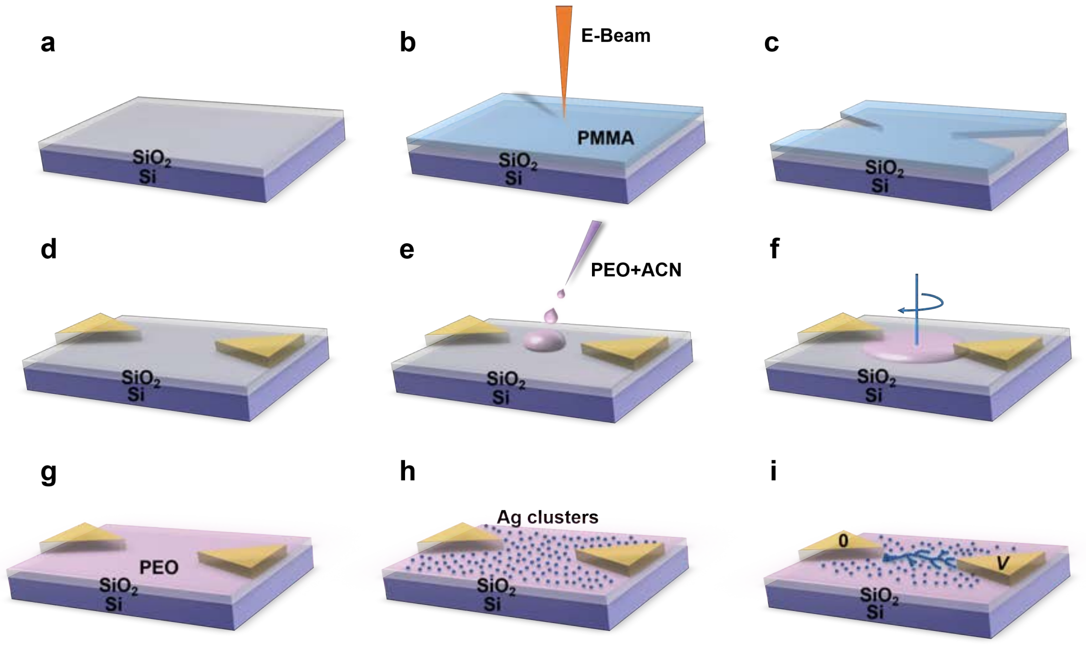

Supplementary Figure 1 | Basic fabrication process of Ag nanocluster based multi-agent evolutionary network system. a, Thermal oxidation method was used to grow 300nm thick

silicon oxide on silicon wafer. Water molecules absorbed on SiO_2 surface were expelled by heating the substrate at 170 °C for 10 min. **b**, PMMA (950,000 g/mol) was spin-coated onto the SiO_2 surface with a spin rate of 3000 r/min for 1 min. The sample was subsequently heated at 170 °C for 3 min to harden the PMMA film. Designed pattern was formed on the PMMA film with electron-beam lithography. **c**, IPA/MIBK (volume ratio: 3:1) was used to develop the pattern for 1 min, followed by fixing the sample in IPA for 30 s. **d**, 1 nm Ti and 40 nm Au were successively deposited by electron beam evaporation and lift-off process was used to form the final electrodes. **e**, Dropping of the polymer electrolyte solution. **f**, Acetonitrile and water molecules were removed from the electrolyte by high-speed spin coating, and thus **g**, the solid polymer electrolyte with uniform thickness was formed. **h**, Electron beam evaporation was used to form Ag nanoclusters partly incorporating into the polymer electrolyte. **i**, Ag conductive filament is connected between the two terminals due to the self-organized evolution of Ag nanoclusters under applied voltage bias.

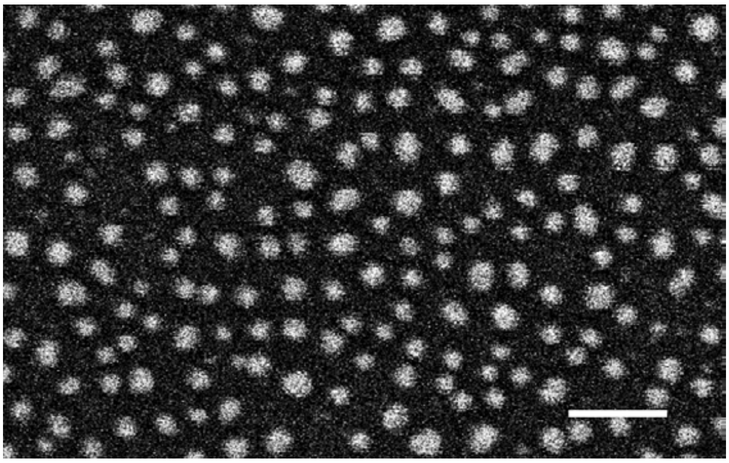

Supplementary Figure 2 | The micro morphology characterization of Ag nanoclusters after device fabrication using SEM technique. Ag nanoclusters are partly incorporated into the solid PEO electrolyte material. Scale bar: 50 nm.

Characterization of the micro-morphology of devices by AFM

In this article, scanning electron microscope (SEM) was mainly used to characterize the microscopic morphology of devices in pursuit of high resolution. In fact, there are many other

feasible ways to achieve the same goals. Here, the surface morphology of sample was characterized by the peakforce tapping mode of atomic force microscope (AFM). Supplementary Fig. 3 gives some results of morphological characterization using AFM.

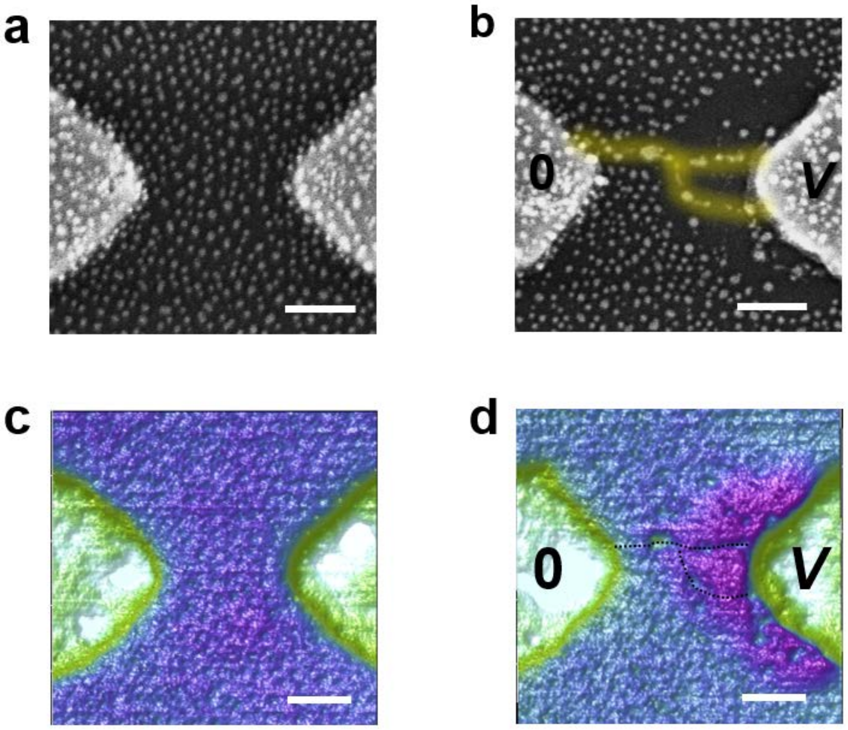

Supplementary Figure 3 | AFM characterization of the micro-topography of device surface. SEM images of two-terminal device **a**, before and **b**, after electrical stimulation, which have been shown in Fig. 1c and 1d, respectively. Scale bar: 100 nm. **c**, AFM image of two-terminal device before memristive switching, corresponding to the SEM image in (**a**). Scale bar: 100 nm. **d**, AFM image of two-terminal device after applying the voltage bias V, corresponding to the SEM image in (**b**). The conductive filament shown in (**d**) is marked with dashed lines as guides for the eyes. Scale bar: 100 nm. The applied voltage *V* was 25 V.

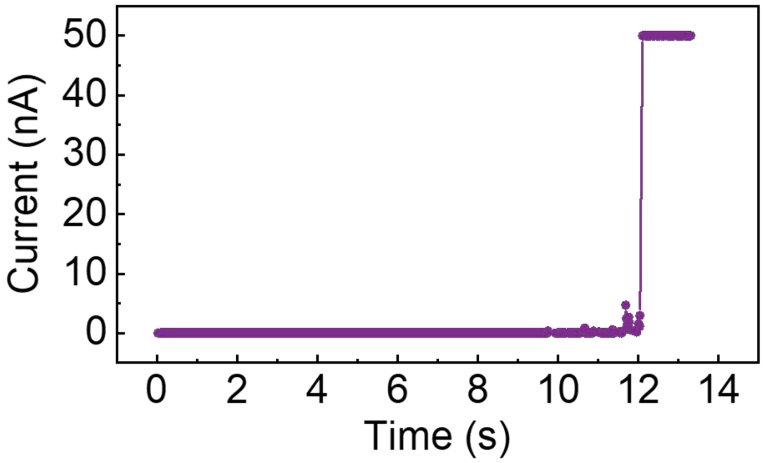

Supplementary Figure 4 | Time-dependent current measurement curve (*I-t*) from the "*V*" terminal corresponding to the Fig. 1(d). The constant voltage bias V = 25 V was applied between the two terminals of device and we can see a significant increase of current at $t \approx 12$ s, indicating the conductive filament is connected between the terminals.

Monte Carlo simulation

In this work, the transport of Ag^+ ions on PEO surface under various electrodes and applied voltages was calculated by kinetic Monte Carlo simulation using Matlab. The simulation process is schematically illustrated in Supplementary Fig. 5. Firstly, the positions of electrodes were initialized according to experimental conditions, and the Ag clusters were randomly generated on the surface. Subsequently, the electrical field distribution was calculated by Kirchhoff's law. During the simulation, each lattice points represents a node and every node is connected with 8 neighbor nodes. When both of the neighbor lattice points were occupied by metal materials (Ag clusters or electrodes), the connected resistance would be set to a low value; otherwise, the connected resistance would be set to a high value.

The boundary condition is that when the edge is an electrode, the potential is the applied voltage, while the insulating boundary would be set to open state. Obviously, internal potentials could be calculated by node analysis, and the electric filed and current could be derived from potentials. Subsequently, the probability of Ag ionization and hopping to unoccupied lattice points could be calculated by Equation (S1) based on Ag distribution and electric fields:

$$p = f \cdot \mathrm{e}^{\frac{-U+nq\Delta V}{k_B T}} \qquad \text{(S1)}$$

Here, p is the ionization-hopping possibility, f is the hoping attempt frequency, U is the hopping barrier between the lattice, nq is Ag^+ ion's charge, ΔV is the potential difference of the lattice, k_B is Boltzmann's constant, T is the absolute temperature. According to the hopping probability, the Ag^+ ion transport could be calculated by Monte Carlo method, where a random number matrix can be generated and compared with the hopping possibility of 8 directions for each lattice so that the next state of Ag distribution can be determined. Finally, the potential would be calculated by the new Ag distribution, followed by simulating Ag^+ ion transport. These processes would be repeated until a conductive filament is formed between the electrodes.

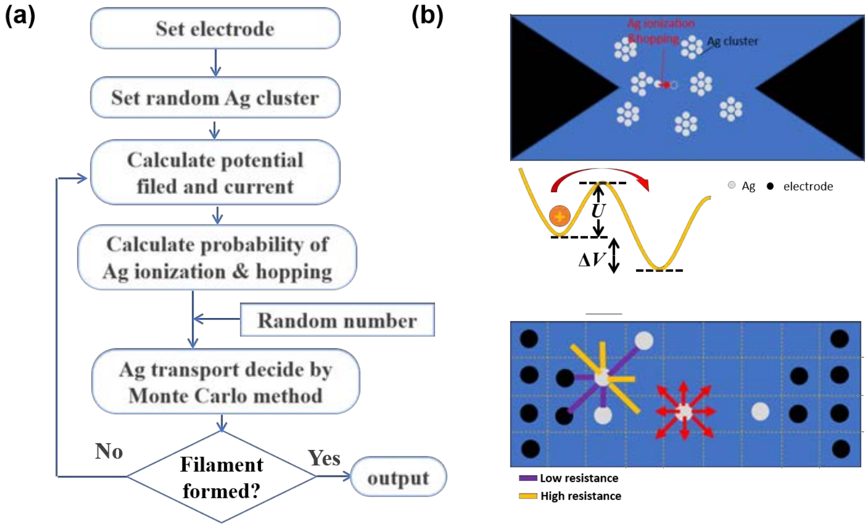

Supplementary Figure 5 | Schematic of the kinetic Monte Carlo simulation process. a, Flow diagram of the simulation process. **b**, The considered physical process and basic calculation method using the resistance network approach in the simulation.

The effect of compliance current on the morphology of conductive filaments

Voltage bias V was applied to the terminals in the two-terminal devices with ~200 nm gap distance under different conditions of compliance current (CC) to investigate the effect of compliance current on the conductive filaments. Supplementary Fig. 6 shows the SEM images of device after electrical stimulation and the corresponding time dependent current measurements (I–t) with different compliance currents (50 nA, 100 nA, 200 nA and 300 nA). We can see that when the same voltage bias V was applied, larger compliance current led to stronger conductive filament and more obvious branching expansion effect. For one thing, the

conductive filament will be too weak to be characterized if the compliance current is too small. For another thing, too large compliance current leads to severe Joule-heating effect which will bring some negative effects such as uncontrollable connection of conductive filaments and increased power consumption. Therefore, it is very important for us to select the appropriate compliance current during the electrical stimulation for obtaining the correct result. In this work, different values of compliance current are selected for different physical structures. For the basic modulation units and parallel structures, a compliance current between 10-50 nA is adopted in most cases. For the problems containing serial structures, larger compliance current of 0.3-10 μA is usually needed to provide larger driving force, due to the generally longer gap distance in serial structures.

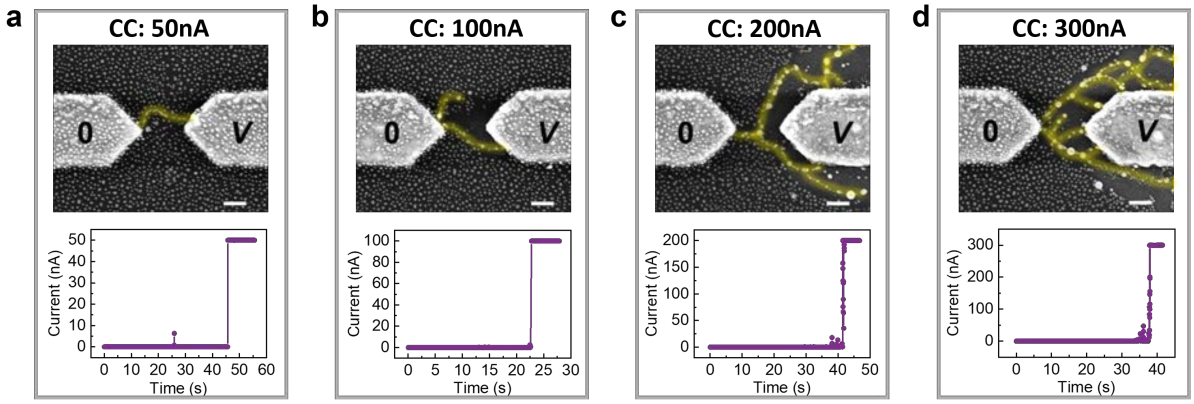

Supplementary Figure 6 | SEM images of the two-terminal devices with ~200 nm gap after memristive switching and the corresponding time dependent current measurements (*I*–*t*) under different compliance current (CC): a, 50 nA; **b**, 100 nA; **c**, 200 nA and **d**, 300 nA. Under the same condition of applied voltage bias *V* (15 V), conductive filament is finally connected between the two terminals and larger compliance current led to stronger filament with more branches. Scale bar: 100 nm.

Pinched hysteresis loops and memristive effect by *I-V* measurements

To study the memristive switching characteristic of our devices, positive and negative voltage sweep were alternately applied to collect the electrical data. Supplementary Fig. 7 shows the forming and subsequent several *I*–*V* sweep curves of a two-terminal device with ~100 nm gap. The repeatable *I-V* curves under 400 alternating sweep cycles are given in Supplementary Fig.

8, indicating the MAEN devices can be stably operated. It can be seen that higher threshold voltage (> 5 V) is needed to drive the initial electrochemical reaction and migration of Ag nanoclusters to form the conductive filament during the forming process. The device spontaneously relaxed back to the off state after removing the voltage bias due to the filament rupture facilitated by interfacial energy minimization with diffusion mechanism (Supplementary Ref. S2). The pinched hysteresis loops from the repeatable *I-V* sweep measurements confirm the memristive nature of our devices.

The MAEN device shows threshold switching characteristics due to the spontaneous dissolution of conductive filament driven by the interfacial energy minimization. Therefore, the device will relax back to the off state after removing the electrical stimulation. Although the continuous filament is broken into discrete clusters, they do not fully recover to their original positions. As a result, the threshold voltages in the subsequent switching processes are significantly reduced, and a new filament can be easily connected based on the previously incompletely ruptured filament. For example, when a small voltage bias (2 V) was applied onto a pristine MAEN device with 100 nm gap, there was no switching within 20 s (Supplementary Fig. 9a). Once a large voltage bias (10 V) was applied, a conductive filament can be formed between the two terminals, along with an obvious increase in current at $t \approx 2.5$ s (Supplementary Fig. 9b). Although the device spontaneously returned to off state after removing the bias, the application of a small voltage bias of 2 V has switched the device to on state again within 1 s (Supplementary Fig. 9c), since the incompletely ruptured filament can be recovered by a smaller driving force. The device can maintain the on-state for a long time at this small voltage bias, i.e. >500 s in Supplementary Fig. 9c. The structure stability of conductive filament can be further tuned by decreasing the gap distance to limit interfacial energy driven surface diffusion. In addition, a smaller gap will be beneficial for the decrease of threshold voltage and solution time, thus saving the computational costs.

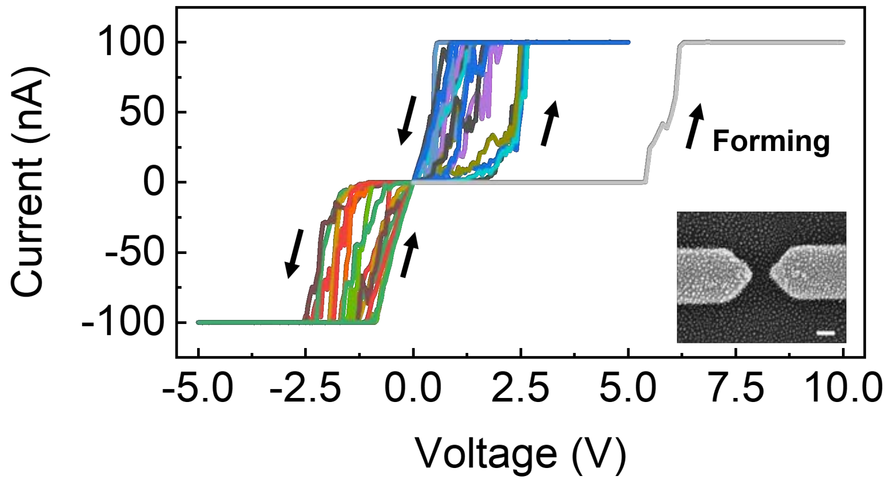

Supplementary Figure 7 | The forming and repeatable *I–V* sweep curves of two-terminal device with ~100 nm gap. A threshold voltage of ~5 V was applied for initial forming process (the gray curve). Subsequently, repeatable *I–V* measurements were performed with alternating positive and negative voltage sweeps, showing repeatable pinched hysteresis loops from off-state to on-state with memristive effect. The inset shows the SEM image of the tested device morphology before memristive switching, scale bar: 100 nm.

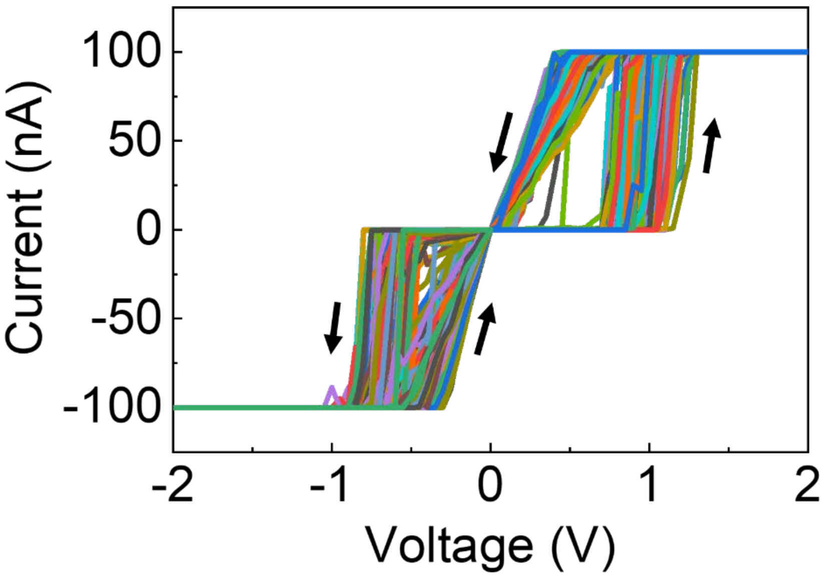

Supplementary Figure 8 | Repeatable *I-V* curves of two-terminal device with 100 nm gap under 400 alternating sweep cycles. The device can be repeatably switched under the alternating positive and negative voltage sweeps.

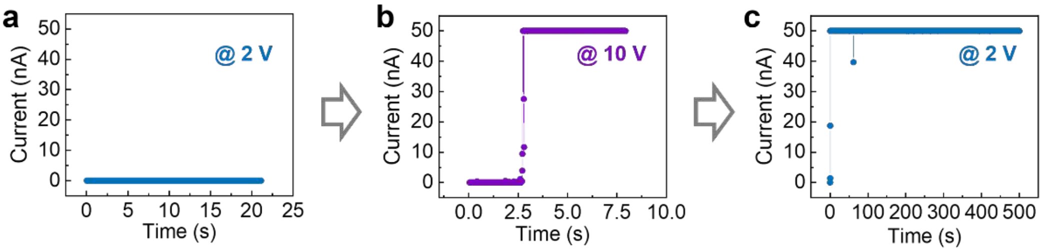

Supplementary Figure 9 | The electrical memory effect due to the incomplete rupture of conductive filament. a, Time-dependent current measurement when a small voltage bias (2 V) was applied on the two-terminal device with 100 nm gap. There is no obvious change for the current within 20 s. **b**, Time-dependent current measurement on the device in (a) under a larger voltage bias of 10 V for the forming process. The current jumped at t ≈ 2.5 s, indicating a connection of conductive filament between the terminals. **c**, Time-dependent current measurement under a small voltage bias (2 V) after forming process in (b). The filament was recovered rapidly with 1 s, and the device can maintain its on-state for a long time under this small voltage bias.

The dynamic evolution process in basic modulation units using MC simulation

Supplementary Fig. 10 (11) gives the dynamic evolution of the Ag atoms/clusters and corresponding electric field distribution in the distance (voltage) modulation unit during the process of Monte Carlo simulation. We note that consistent with the field-driven nature of Ag cluster evolution process, the most visible cluster evolution occurs at places with the highest electric field intensity. The conductive filament is finally formed along the path with the shortest gap distance (Supplementary Fig. 10, distance modulation unit) or highest voltage bias (Supplementary Fig. 11, voltage modulation unit), which completely reproduces our experimental results (Fig. 1j and Fig. 1m).

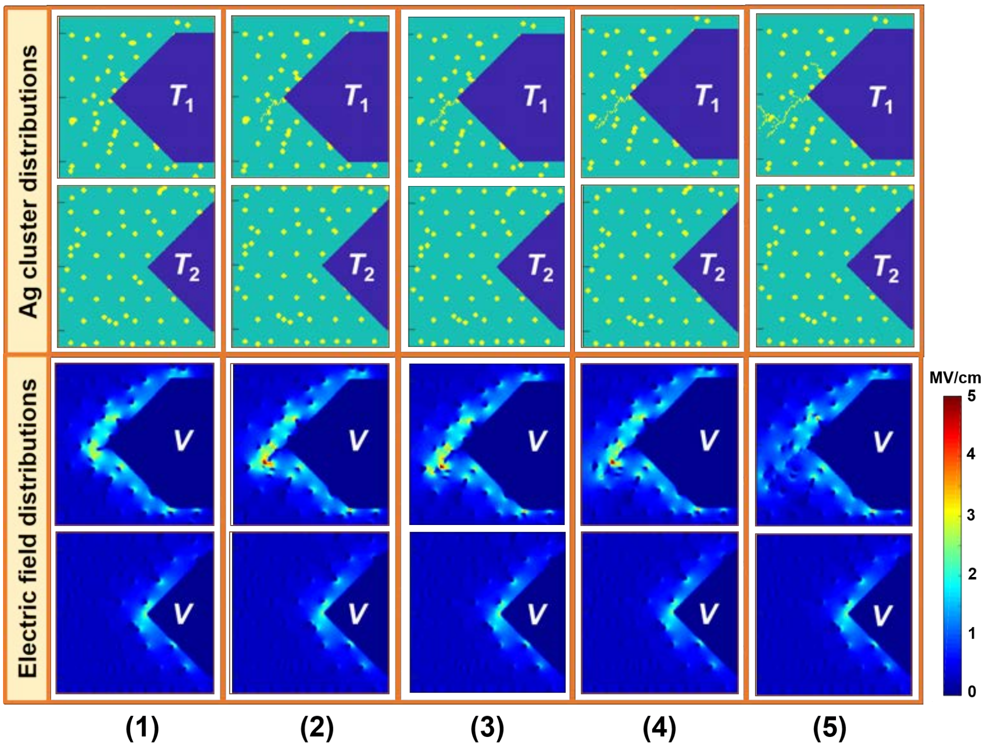

Supplementary Figure 10 | The physical evolution processes of the distance modulation unit using Monte Carlo simulation: Ag atom/cluster evolution (upper panels) and corresponding electric field distributions (bottom panels). The conductive filament is finally connected along the shortest path.

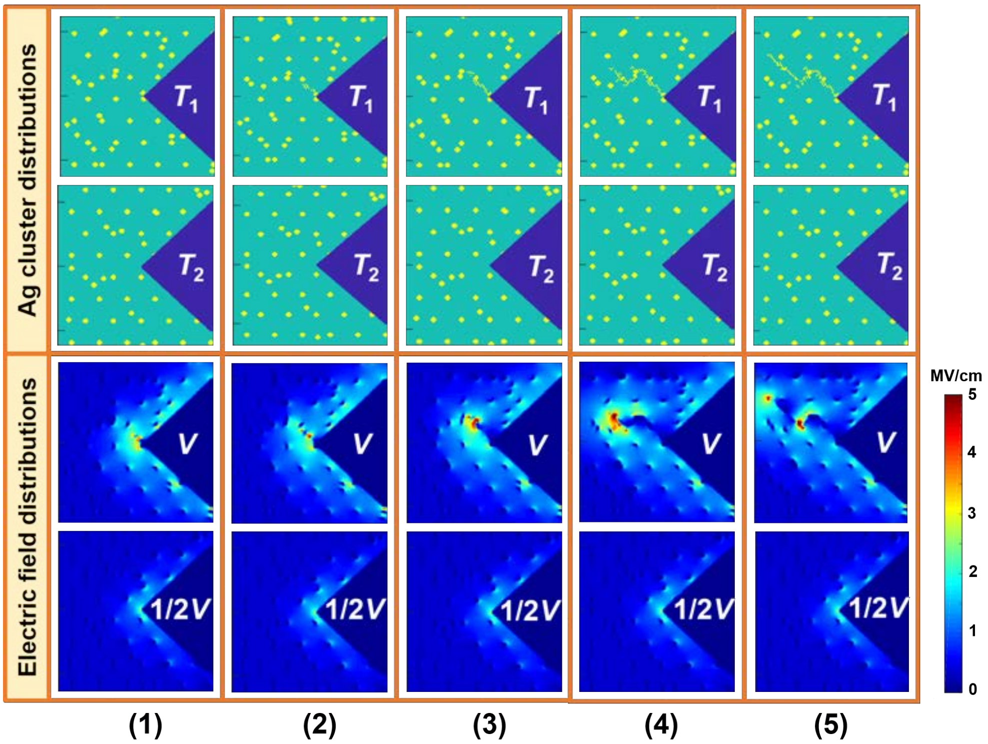

Supplementary Figure 11 | The physical evolution processes of the voltage modulation unit using Monte Carlo simulation: Ag atom/cluster evolution (upper panels) and corresponding electric field distributions (bottom panels). The conductive filament is finally connected along the path corresponding to the highest voltage bias.

Proof of the gap equivalence in the voltage modulation unit

In Fig. 1m, the voltage bias V representing smaller weight was applied between the terminals T_1 and T_3 while $1/2V$ representing larger weight was applied between the terminals T_2 and T_3. Conductive filament is finally connected between the terminals T_1 and T_3, which corresponds to the selection of edge with smaller weight following the optimal solution principle. In order to prove the equivalence of gap in the voltage modulation unit for solving problems, we interchanged the potential applied to the terminals T_1 and T_2. Supplementary Fig. 12 shows that conductive filament establishes connection between the terminals T_2 and T_3 when the voltage bias V representing smaller weight is applied between the terminals T_2 and T_3. Combined with the result shown in Fig. 1m, we can conclude that all the gaps are initially equivalent in the voltage modulation unit, and the connection path of conductive filament is exclusively determined by the voltage bias.

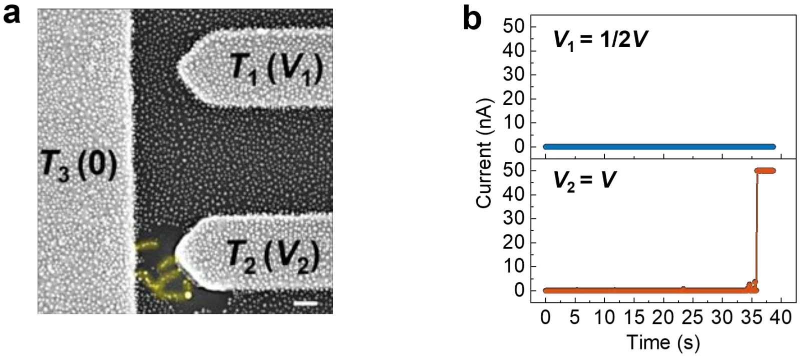

Supplementary Figure 12 | Proof of the gap equivalence in the voltage modulation unit by interchanging the potential applied to the terminals T_1 and T_2 of device structure in Fig. 1m. a, SEM image of device morphology after resistive switching. The distance of two gaps were ~200 nm. The voltage bias $1/2V$ was applied between the terminals T_1 and T_3 while the voltage bias V was applied between the terminals T_2 and T_3. Conductive filament is finally connected between the terminals T_2 and T_3. Scale bar: 100 nm. **b**, Time-dependent current

measurement from the terminals T_1 (blue curve) and T_2 (orange curve). The applied V was 30 V.

Hybrid distance-voltage modulation unit

Supplementary Fig. 13 gives the initial state of an as-fabricated device corresponding to the Fig. 1o-q before memristive switching and some repeated experiment verifications. The consistent results suggest the reliability of basic modulation units for solving problems.

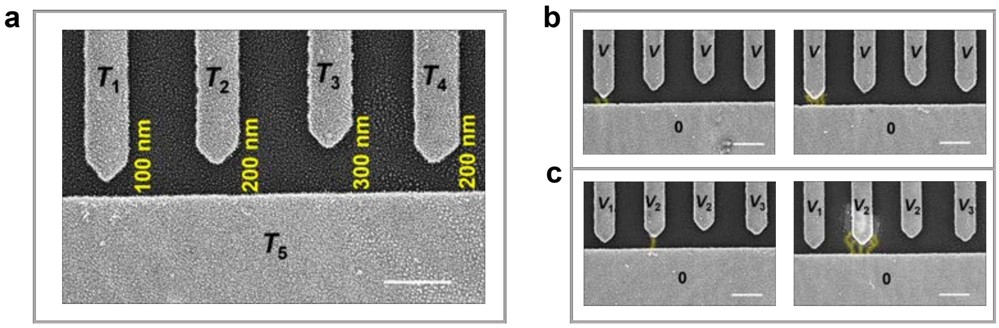

Supplementary Figure 13 | The hybrid distance-voltage modulation unit. a, SEM image of the device morphology before memristive switching. The designed gap distance from left to right is 100 nm (T_1-T_5), 200 nm (T_2-T_5), 300 nm (T_3-T_5) and 200 nm (T_4-T_5), respectively. Scale bar: 500 nm. **b**, SEM images of repeated experiment results corresponding to the voltage bias scheme in Fig. 1o. The applied voltage bias V was 20 V. Scale bar: 500 nm. **c**, SEM images of repeated experiment results corresponding to the voltage bias scheme in Fig. 1p. The applied voltage biases V_1, V_2 and V_3 were 15 V, 25 V and 20 V, respectively. Scale bar: 500 nm.

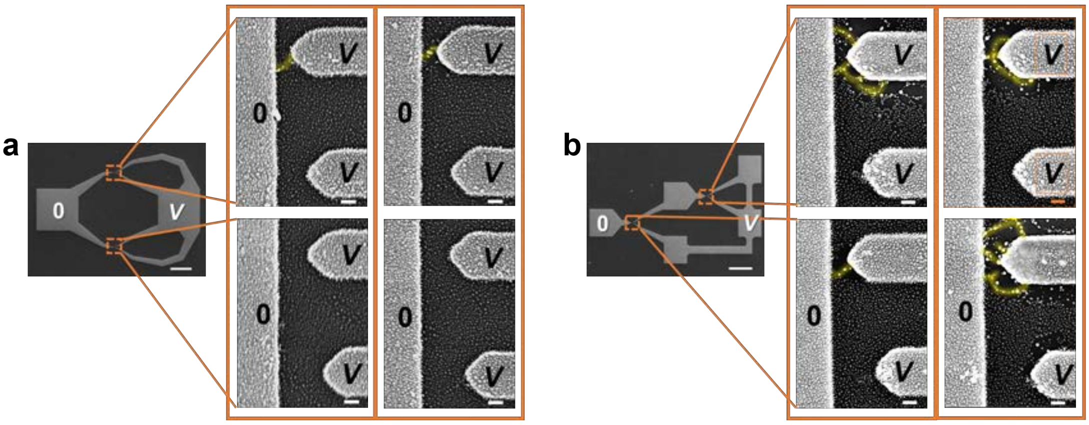

Supplementary Figure 14 | Repeated experiment results of integration schemes: a, parallel

structure in Fig. 2b and **b**, serial structure in Fig. 2e. In order to prove that the experiment results of expansion structure shown in Fig. 2 were not the accidental random phenomena, multiple devices were tested and consistent solution results were reliably obtained. The left panel in (**a**) shows the global configuration of parallel structure and corresponding voltage bias scheme, scale bar: 50 μm; The middle panel and the right panel demonstrate the connectivity patterns of conductive filament after memristive switching consistent with the result in Fig. 2b, scale bar: 100 nm. The applied voltage V was 17.5 V. The left panel in (**b**) shows the global configuration of serial structure and corresponding voltage bias scheme, scale bar: 100 μm; The middle panel and the right panel demonstrate the connectivity patterns of conductive filament after memristive switching consistent with the result in Fig. 2e, scale bar: 100 nm. The applied voltage V was 15 V.

Intrinsic stochastic nature of the conductive filament growth

In the hybrid distance-voltage modulation unit shown in Supplementary Fig. 13a, two solution results were observed in our experiments under the voltage bias scheme shown in Supplementary Fig. 15: the conductive filament is only formed between the terminals T_4 and T_5 (Supplementary Fig. 15a, case 1); the conductive filament is both formed between the terminals T_2 and T_5, and between the terminals T_4 and T_5 (Supplementary Fig. 15b, case 2). The different connectivity results were also observed in the integration structures of basic modulation units. Supplementary Fig. 16 demonstrates three connectivity patterns observed in the parallel structure composed of two identical voltage modulation units under a voltage bias scheme: the conductive filament is only formed between the terminals T_2 and T_3 (Supplementary Fig. 16, case1); the conductive filament is only formed between the terminals T_4 and T_6 (Supplementary Fig. 16, case2); the conductive filament is both formed between the terminals T_2 and T_3, and between the terminals T_4 and T_6 (Supplementary Fig. 16, case3). The diverse connectivity patterns are essentially derived from the stochastic nature of the filament growth process (Supplementary Ref. S3 and S4) due to the physiochemical processes involved and the inhomogeneity of cluster distribution.

a

b

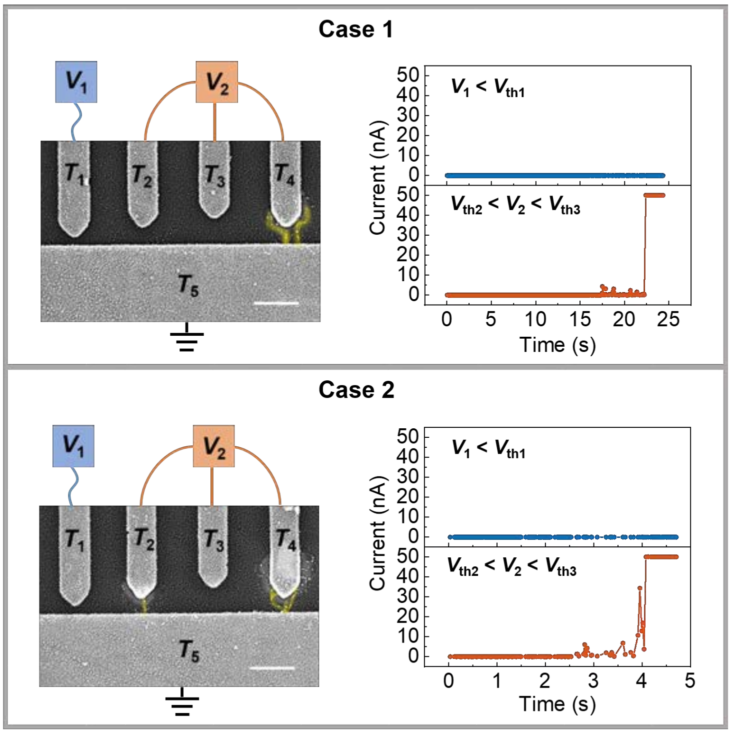

Supplementary Figure 15 | Stochastic nature of conductive filament growth in basic modulation unit. The device structure before electrical stimulation is shown in Supplementary Fig. 13a. The voltage bias V_1 (10 V) was applied between the terminals T_1 and T_5 while the voltage bias V_2 (25 V) was applied between the terminals $T_{2(3,4)}$ and T_5. **a**, One case of the observed connectivity pattern where the conductive filament is formed between the terminals T_4 and T_5. The left panel shows the SEM image of devices structure after memristive switching and the right panel shows the corresponding time-dependent current curve. Scale bar: 500 nm. **b**, The other case of the observed connectivity pattern where the conductive filament is both formed between the terminals T_2 and T_5, and between the terminals T_4 and T_5. The left panel shows the SEM image of devices structure after memristive switching and the right panel shows the corresponding time-dependent current curve. Scale bar: 500 nm.

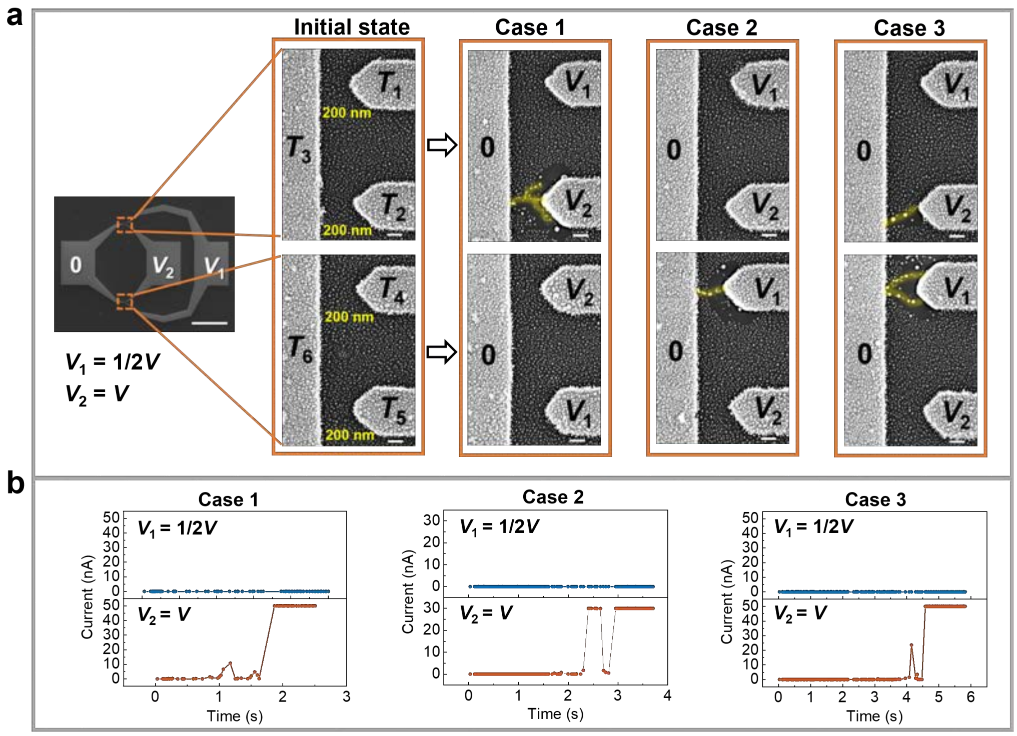

Supplementary Figure 16 | Stochastic nature of conductive filament growth in the parallel structure of basic modulation units. a, SEM images of parallel structure composed of two identical voltage modulation units. The left panel shows the global structure and the corresponding voltage bias scheme. Scale bar: 100 μm. The panel marked with "Initial state" shows the device morphology before electrical stimulation. The designed distance of each gap was 200 nm. Scale bar: 100 nm. The panels marked with "case 1(2, 3)" show the SEM images of different connectivity patterns under the identical voltage bias scheme. Scale bar: 100 nm. **b**, Time-dependent current measurements corresponding to case 1(2, 3) of connectivity patterns in (**a**). The applied voltage bias V was 20 V.

Fabrication of the metal islands

Supplementary Fig. 17 shows the fabrication of metal islands. Same materials and lithography conditions were used to fabricate the metal islands and the metal electrodes in this article. Therefore, the metal islands can be prepared in the same steps for the preparation of metal electrodes by following the basic process shown in Supplementary Fig. 1. If the lithography condition of metal islands does not match that of metal electrodes, twice pattern processes are respectively needed.

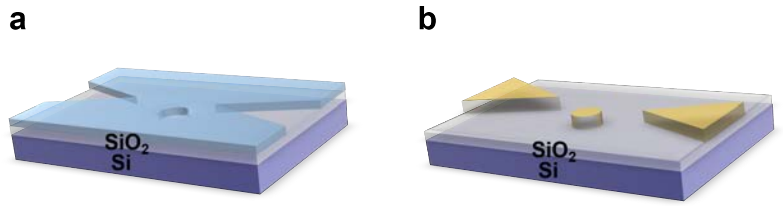

Supplementary Figure 17 | Fabrication of the metal islands used for intermediate node representation and attractive modulation. In this work, the fabrication of metal islands was simultaneously carried out with the fabrication of metal electrodes shown in Supplementary Fig. 1. **a**, The PMMA pattern of metal electrodes and metal islands after electron beam lithography and development. **b**, Final pattern of metal electrodes and metal islands after electron beam evaporation and lift-off process.

Monte Carlo simulation of evolving process in the device with metal islands

In order to further prove the modulation effect of metal islands on the connectivity pattern of conductive filament, Monte Carlo simulation was carried out to study the physical process involved in the evolution of system. Supplementary Fig. 18 shows the evolution of Ag atom/cluster distributions (upper panels) and corresponding electric field distributions (bottom panels) in the device in Fig. 3f over time, obtained from a kinetic Monte Carlo simulation. We can clearly see that the metal islands between the terminals effectively enhance the electric field intensity between the metal islands with each other, and between the metal islands and the terminals, leading to the competitive conductive filament growth along the arrangement direction of upper two metal islands. The final connectivity pattern of conductive filament in the MC simulation is consistent with the experimental results in Fig. 3g.

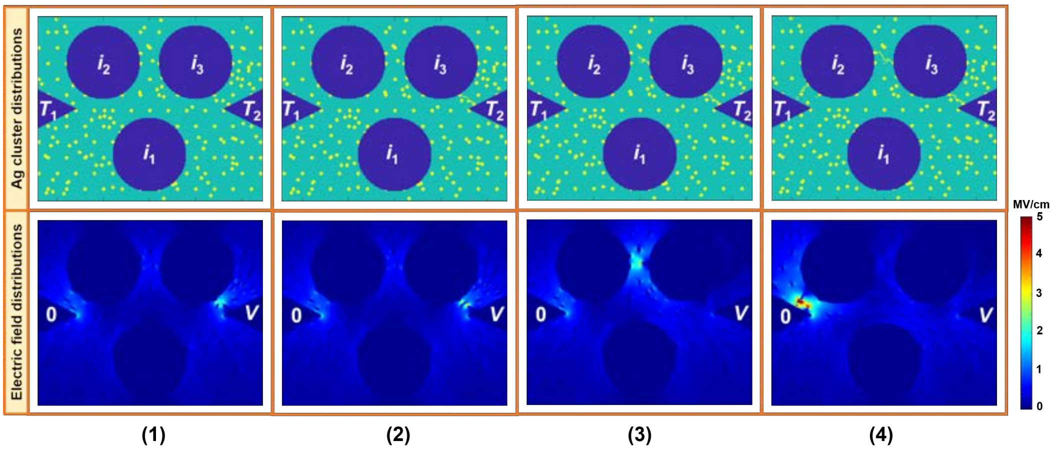

Supplementary Figure 18 | The physical evolution processes of the device with metal islands using Monte Carlo simulation: Ag atom/cluster evolution (upper panels) and corresponding electric field distributions (bottom panels). The conductive filament is finally connected between the terminals T_1 and T_2 by way of the islands i_2 and i_3 following the principle of the highest electric field.

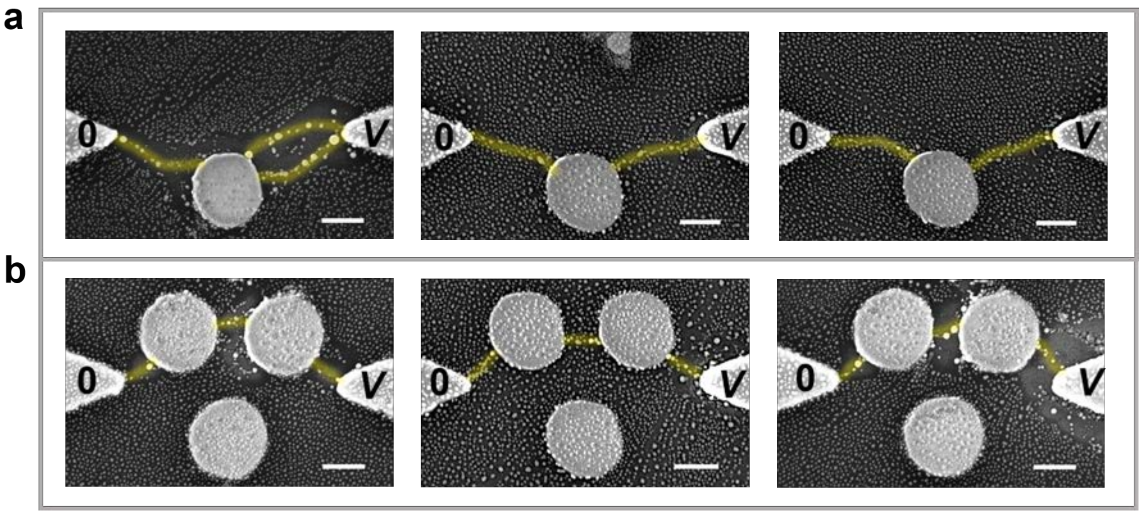

Supplementary Figure 19 | Repeated results of connectivity patterns obtained from multiple devices with metal islands. a, SEM images of the devices with the structure shown in Fig. 3b after memristive switching, consistent with the results shown in Fig. 3c. The applied voltage bias *V* was 25 V. Scale bar: 200 nm. **b**, SEM images of the devices with the structure shown in Fig. 3f after memristive switching, consistent with the results shown in Fig. 3g. The applied voltage bias *V* was 20 V. Scale bar: 200 nm.

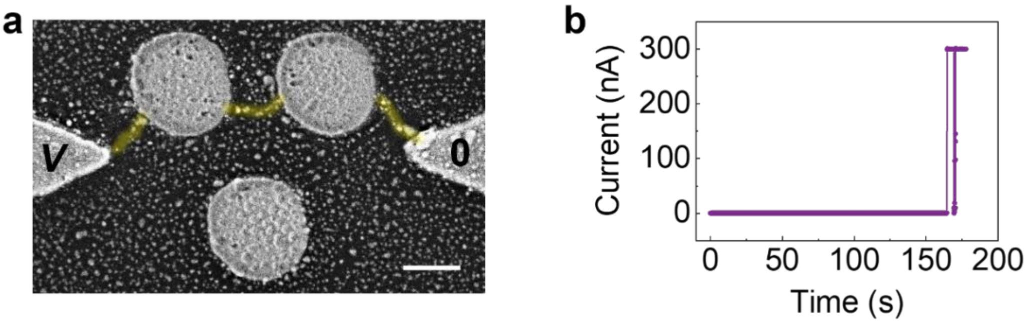

Supplementary Figure 20 | Solution result with opposite voltage bias in Fig. 3g (i.e. the voltage bias *V* is applied to the terminal T_1 while the terminal T_2 was grounded). The

definition of terminals is shown in **Fig. 3f**. a, SEM image of the filament morphology after memristive switching. Ag nanoclusters physically evolving along the metal islands i_2 (N_4) and i_3 (N_5) still won the competition, and the conductive filament established connection between the terminals T_1 (N_1) and T_2 (N_2) by way of the metal islands i_2 (N_4) and i_3 (N_5) under the applied voltage bias V (35 V). Scale bar: 200 nm. **b**, *I-t* curve corresponding to the experiment in (**a**).

The effect of terminal position on the connectivity pattern of conductive filament

In addition to the metal islands, the terminal position can also effectively regulate the electric field distribution to affect the final connectivity pattern of conductive filament, and thus the flexibility of graph structure mapping can be further improved. The completely different graph structure information can be expressed by simply regulating the terminal position of devices. For the graph structure shown in Supplementary Fig. 21a, the problem to be solved is to find the shortest path between the nodes N_1 and N_2. This problem can be mapped into the device configuration shown in Supplementary Fig. 21b where the terminal T_2 on the right side of device in Fig. 3f is moved to the upper side. The change of terminal position directly affects the electric field distribution, thus the connectivity pattern of conductive filament is accordingly modified. From the Supplementary Fig. 21c we can see that the conductive filament is connected between the terminals T_1 (N_1) and T_2 (N_2) by way of the metal island i_2 (N_4) after electrical stimulation. The connection path ($d_6 + d_9$) corresponded to the selection of edges with the smallest total weight, i.e. $N_1 \overset{1}{\leftrightarrow} N_4 \overset{1}{\leftrightarrow} N_2$ (Supplementary Fig. 21a).

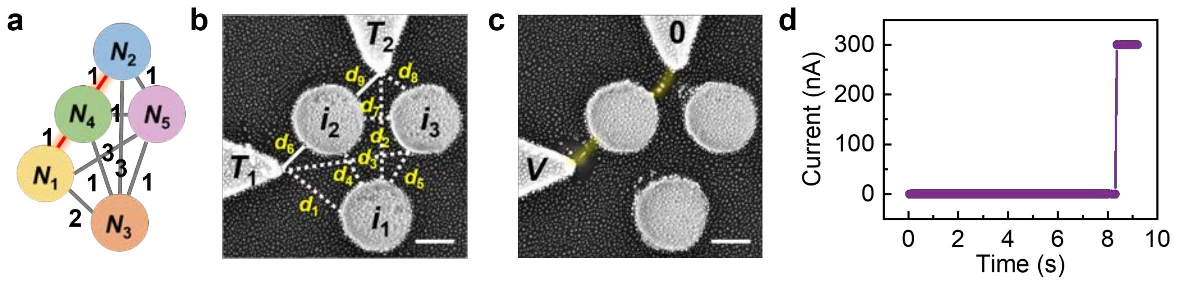

Supplementary Figure 21 | The influence of the terminal position on the connection path of conductive filament. a, Schematic illustration of a graph structure with three intermediate nodes N_3, N_4 and N_5 between the nodes N_1 and N_2. **b**, SEM image of device morphology mapping with the graph structure in (**a**). The terminal T_2 in Fig. 3f is moved to the upper side

while other settings remained unchanged. Scale bar: 200 nm. **c**, SEM image of the device in (**b**) after memristive switching. The connection path in Fig. 3g is changed accordingly due to the adjustment of T_2 terminal position. The applied voltage bias V was 20 V. Scale bar: 200 nm. **d**, Time-dependent current measurement corresponding to the resistive switching process. The connection of conductive filament between the terminals T_1 and T_2 by way of the metal island i_2 is driven by the self-organized evolution of Ag nanoclusters under the electrical stimulation, leading to an obvious increase of current in the (**d**).

The reusability of MAEN system

The spontaneous diffusion dynamics of conductive filament driven by interfacial energy minimization between the Ag nanoclusters and dielectrics (Supplementary Ref. S2 and S5) causes electrical disconnection between the electrode terminals after removing the external biasing, which provides substrate for reusing the same MAEN device for the solution of problems. Supplementary Fig. 22 shows a consecutive sequence of operations on the same MAEN system with 4 terminals and 3 metal islands. For each step, the conductive filament can establish connection along the optimal path between the corresponding terminals, and the spontaneous relaxation of particles after removing the biases allows the device to be reused and operate correctly in the next steps. It is worth mentioning that the previous connectivity patterns may have a certain impact on the subsequent solutions. In the future, material systems with large wetting contact angles, such as MgO_x:Ag, SiO_xN_y:Ag and HfO_x:Ag (Supplementary Ref. S2), may be considered. It is reported that the conductive filament may quickly shrink to original Ag nanoclusters driven by interfacial energy in these material systems when the external biasing is removed, so that the reusability of system can be further improved.

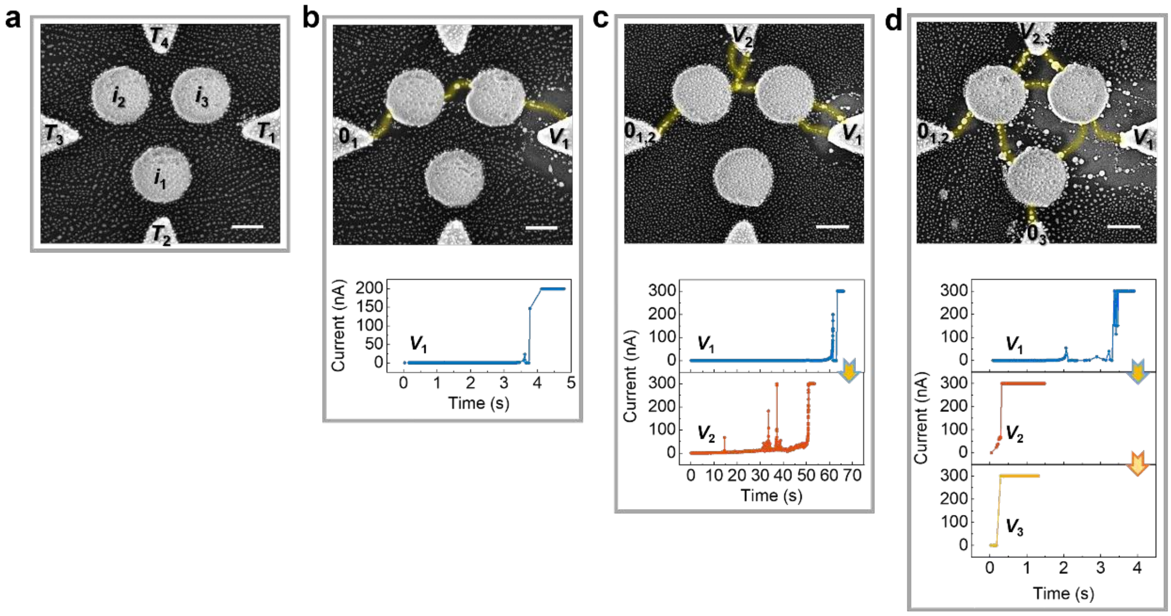

Supplementary Figure 22 | Sequential operations on the MAEN systems with 4 terminals (T_1, T_2, T_3 and T_4) and 3 metal islands (i_1, i_2 and i_3). a, SEM image of the device structure before electrical stimulation. Scale bar: 200 nm. **b**, SEM image of the filament morphology when the voltage bias V_1 (25 V) was applied between the terminals T_1 and T_3. The conductive filament was formed between the terminals T_1 and T_3 by way of the metal islands i_2 and i_3 under the applied voltage bias V_1. Scale bar: 200 nm. **c**, SEM image of the filament morphology when the voltage bias V_1 (25 V) was applied between the terminals T_1 and T_3, followed by a voltage bias V_2 (10 V) between the terminals T_3 and T_4. Correspondingly, the conductive filament was connected between the terminals T_3 and T_4 along the metal island i_2 under the voltage bias V_2 in addition to the connection path shown in (**b**) under the voltage bias V_1. Scale bar: 200 nm. **d**, SEM image of the filament morphology after successively applying three voltage bias schemes (i.e. the voltage bias V_1 (15 V) between T_1 and T_3, the voltage bias V_2 (5 V) between T_3 and T_4 and the voltage bias V_3 (5 V) between T_2 and T_4). Since there exists two optimal path between the terminals T_2 and T_4 under the voltage bias V_3, the conductive filament was both formed by way of the metal islands i_1 and i_2 and by way of the metal islands i_1 and i_3 in addition to the connection path shown in (**c**) under the voltage bias V_1 and V_2. Scale bar: 200 nm. The subscripts of applied voltage bias labelled in the figures represent the order of three voltage bias schemes.

The reconfigurability of MAEN system

A reconfigurable computing system is needed for solving different problems with the same structure. In fact, the MAEN system possesses potential for reconfigurability. Supplementary Fig. 23 gives an example to show that different problems can be solved with the same device structure. We can see that under two different voltage bias schemes, Ag nanoclusters spontaneously align into a connective filament along the optimal path, leading to two different connectivity patterns. In the case of Supplementary Fig. 23a, V and 0 were applied to the neighboring terminals T_1 and T_4 respectively, while the other two terminals (i.e. T_2 and T_3) were biased at $1/2V$. In this case, both distance modulation and voltage modulation are involved. The voltage biases V and $1/2V$ were applied to the paths with equal length "$T_1 \leftrightarrow i_3 \leftrightarrow T_4$" and "$T_3 \leftrightarrow i_2 \leftrightarrow T_4$", constituting the voltage modulation mode. Since the applied voltage bias between T_1 and T_4 is higher, the conductive filament was formed along the path "$T_1 \leftrightarrow i_3 \leftrightarrow T_4$" where the electric field is highest, leading to a significant increase of current from the terminals T_1 and T_4. In another case of Supplementary Fig. 23b, V and 0 were applied to the diagonal terminals T_1 and T_3 respectively, while the other two terminals (i.e. T_2 and T_4) were assigned to $1/2V$. Compared with the previous case, a different pattern connecting the terminals T_1 and T_3 by way of the metal islands i_2 and i_3 (i.e. "$T_1 \leftrightarrow i_3 \leftrightarrow i_2 \leftrightarrow T_3$") was formed. In addition, the graph problem shown in Fig. 3e can also be solved by using the device structure shown in Supplementary Fig. 23, where the original two-terminal device structure (Fig. 3f) is included as a sub-graph. In future research works, we propose that the back gates can be further added to achieve gate modulation. Once the back gates are incorporated, the weight can be represented by the gate control signal, which provides another degree of freedom to effectively regulate cluster evolution and filament growth. By applying a driving bias signal while the control signals representing weight information are applied to the corresponding gate. The gate modulation scheme combines the advantages of distance modulation and voltage modulation schemes, simultaneously possessing high efficiency, high flexibility and high reconfigurability for solution of problems. Supplementary Fig. 24 gives an example of solving a 3×3 maze problem based on the gate modulation unit. Thanks to the same device structure independent of the specific maze, a system mapping the $N \times N$ maze is capable of solving arbitrary $n \times n$ maze problems ($n \leqslant N$), suggesting high reconfigurability of the system.

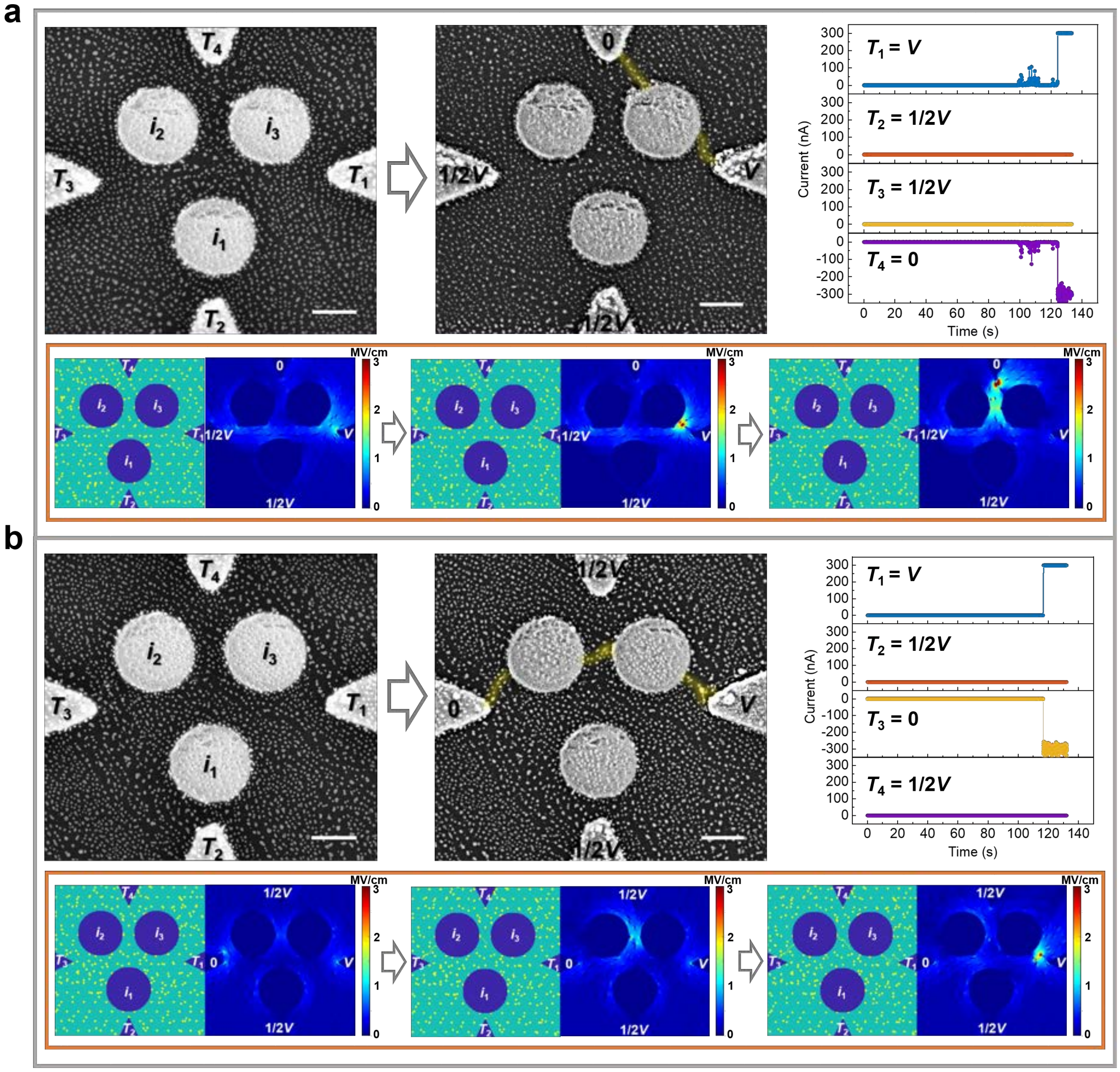

Supplementary Figure 23 | Connectivity patterns under two different voltage bias schemes in the same device structure with 4 terminals (T_1, T_2, T_3 and T_4) and 3 metal islands (i_1, i_2 and i_3). a, One case where V and 0 were respectively applied to the neighboring terminals T_1 and T_4, while the other two terminals (i.e. T_2 and T_3) were biased at 1/2V: SEM images of device morphology before memristive switching (upper left panel, scale bar: 200 nm) and after applying the voltage bias scheme (upper middle panel, scale bar: 200 nm); corresponding time-dependent current measurement from 4 terminals (upper right panel), and Monte Carlo simulation (bottom panels). The applied voltage bias V was 15 V. The conductive filament finally established connection between the terminals T_1 and T_4 by way of metal island i_3. **b**, Another case where V and 0 were respectively applied to the diagonal terminals T_1 and

T_3, while the other two terminals (i.e. T_2 and T_4) were assigned to $1/2V$: SEM images of device morphology before memristive switching (upper left panel, scale bar: 200 nm) and after applying the voltage bias scheme (upper middle panel, scale bar: 200 nm); corresponding time-dependent current measurement from 4 terminals (upper right panel), and Monte Carlo simulation (bottom panels). The applied voltage bias V was 30 V. The conductive filament was finally formed between the terminals T_1 and T_3 by way of metal islands i_2 and i_3.

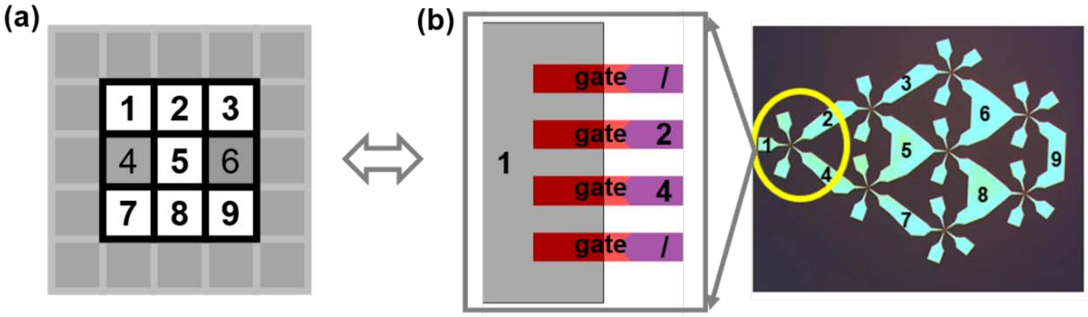

Supplementary Figure 24 | A 3×3 maze problem solved by the gate modulation scheme. **a**, The connectivity of maze. The white grid represents connected and the grey grid represents disconnected. **b**, The MAEN system with topological structure capable of mapping the 3×3 maze by cascading the basic gate modulation units using parallel and serial schemes. The enlarged image on the left exemplifies the gate modulation unit representing the connectivity of grid 1 in (**a**).

The improvement of system generality with unified node shape

In order to demonstrate the potential of system generality, the sharp tip of electrode terminals was replaced by more rounded shape to share a similar geometry with the circular metal islands. From Supplementary Fig. 25 one can see that the connectivity pattern of conductive filaments after changing the terminal shape (Supplementary Fig. 25b) is exactly same as before (Supplementary Fig. 25a) under the same voltage scheme. Two shortest paths were simultaneously founded relying on the self-organized evolution of Ag nanoclusters. The result indicates that the optimal route between each two nodes in the MAEN system may be computed

because they share similar geometry, providing more general and promising application scenarios.

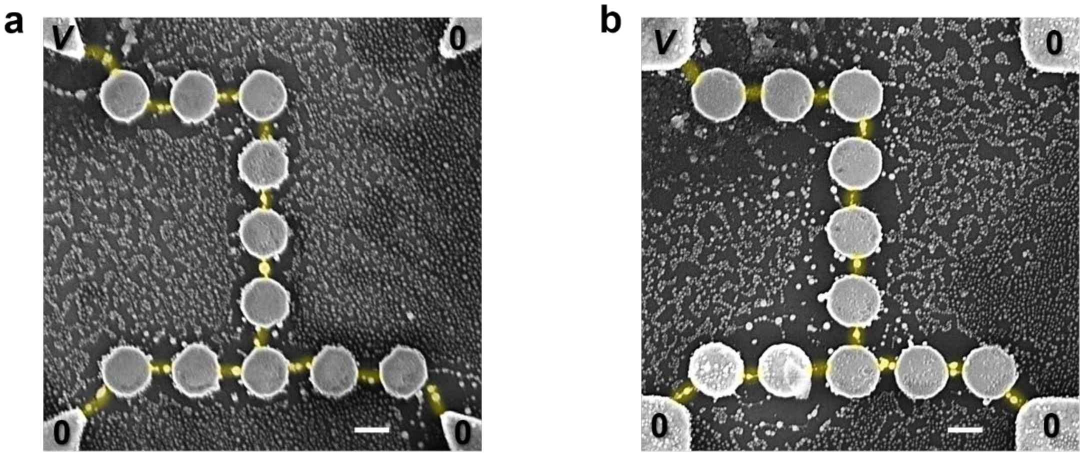

Supplementary Figure 25 | The impact of terminal shape on the connectivity pattern under the same voltage bias scheme. a, SEM image of connection path in the device structure with sharp terminal tips and circular metal islands under the voltage bias scheme. The applied voltage V was 30 V. Scale bar: 200 nm. **b**, SEM image of connection path in the device structure where the sharp terminal tips in (**a**) were replaced by the more rounded terminal tips. The applied voltage V was 30 V. Scale bar: 200 nm. Two shortest paths were founded in (**a**) and (**b**) relying on the self-organized evolution of Ag nanoclusters.

Fabrication of the obstacles in the MAEN

For the fabrication of obstacles with lower Ag^+ ion mobility (Supplementary Fig. 26), dual-beam focused ion beam (FIB) technique was used to bombard the polymer electrolyte material PEO with gallium ions for etching after the manufacturing process shown in Supplementary Fig. 1. The position and shape of etching can be precisely controlled to expose the silicon dioxide with lower Ag^+ ion mobility so that the etched area serves as the obstacle to hinder the migration of Ag^+ ions.

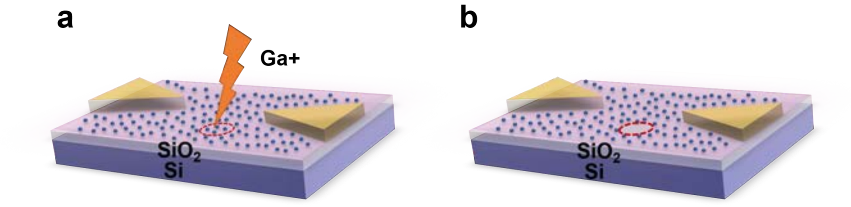

Supplementary Figure 26 | Construction of the obstacles by FIB etching for repulsive modulation. a, Gallium ion beam was used to bombard the sample surface after (**i**) in Supplementary Fig. 1 to etch the polymer electrolyte PEO with high-precision. **b**, Schematic diagram of the sample after being etched. The material in the red circle is silicon oxide and the rest area is still the polymer electrolyte.

Monte Carlo simulation of artificial potential field based on the MAEN

The physical process of artificial potential field evolution in Fig. 4c under the comprehensive control of electric field and ion mobility was further studied using kinetic Monte Carlo simulation. Supplementary Fig. 27 shows the evolution of Ag atom/cluster distributions over time, obtained from the kinetic Monte Carlo simulation. The experimentally observed connection path of conductive filament shown in Fig. 4d was well reproduced.

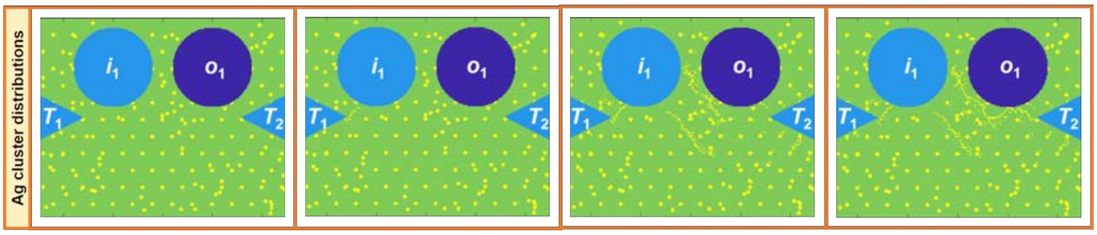

Supplementary Figure 27 | The Ag atom/cluster evolution processes of the artificial potential field hardware using Monte Carlo simulation. The conductive filament adaptively avoids the obstacle o_1 to establish a connection between the terminals T_1 and T_2 by way of the attractor i_1.

The effect of position exchange of attractor i_1 and obstacle o_1 on the solution result

Here, we performed Monte Carlo simulation where the position of attractor i_1 and obstacle o_1 are swapped in the artificial potential field problem shown in Fig. 4f. From Supplementary Fig.

28 one can see that the solution result is not affected since the electric field distribution will be symmetric, leading to the same cluster evolution result. Consistent with the result shown in Fig. 4g, the filament still establishes connection between the terminals T_1 and T_2 through the path without obstacles by way of i_2 (i.e. $T_1 \overset{d_5}{\leftrightarrow} i_2 \overset{d_6}{\leftrightarrow} T_2$).

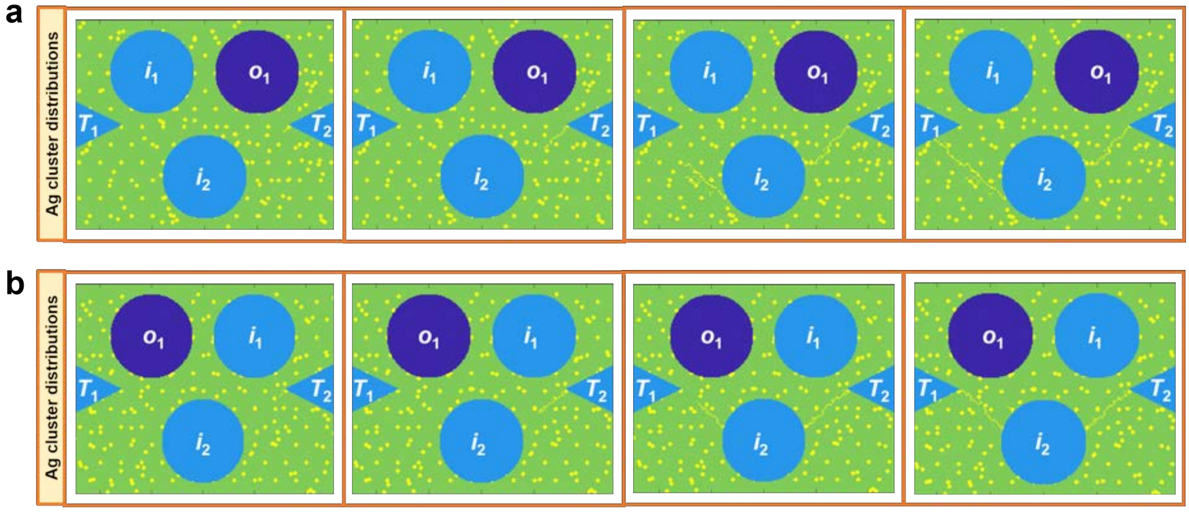

Supplementary Figure 28 | The Monte Carlo simulation of the artificial potential field shown in Fig. 4f before and after swapping the position of attractor i_1 and obstacle o_1. a, The Ag atom/cluster evolution processes in Fig. 4f when the voltage bias was applied between the terminals T_1 and T_2. The conductive filament was connected along the path $T_1 \overset{d_5}{\leftrightarrow} i_2 \overset{d_6}{\leftrightarrow} T_2$, which is consistent with the experiment result shown in Fig. 4g. **b**, The Ag atom/cluster evolution processes after swapping the position of attractor i_1 and obstacle o_1 in Fig. 4f. The conductive filament still establishes connection between the terminals T_1 and T_2 through the path without obstacles by way of i_2.

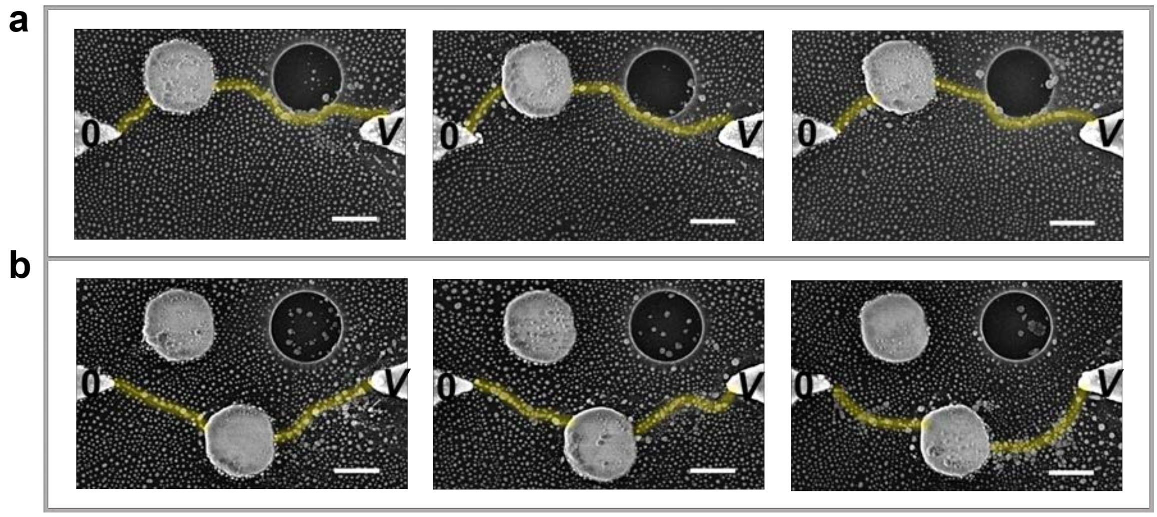

Supplementary Figure 29 | The repeated results of connectivity patterns in the constructed artificial potential field. a, SEM images of the devices with the structure shown in Fig. 4c after memristive switching, consistent with the results shown in Fig. 4d. The applied voltage bias *V* was 40 V. Scale bar: 200 nm. **b**, SEM images of the devices with the structure shown in Fig. 4f after memristive switching, consistent with the results shown in Fig. 4g. The applied voltage bias *V* was 30 V. Scale bar: 200 nm.

MAEN circuit modules for efficient writing/reading

In this work, the solution result represented by the connectivity pattern of the filament was mainly obtained by SEM observations, and the time-dependent current measurement was used as an auxiliary readout method, since the detailed connectivity pattern may not be completely reflected through the limited number of probes in the testing probe stations. To achieve efficient writing/reading when the problems to be solved contain multiple inputs/outputs, a dedicated circuit platform for electrical measurements can be developed for the MAEN system. Supplementary Fig. 30 depicts a schematic diagram of the reading and writing periphery circuits. The module "CTRL" is the controller that sends control signals to decide corresponding operations, such as the terminals to be addressed and the voltage bias to be applied. The multiplexer "MUX" receives control signal from "CTRL" and selects the voltage bias that corresponds to the terminals. The signal converter "ADC & DAC" is used to input and read the analog electrical signal. We should point out that the SEM observation in this work is to demonstrate the correct solution of the problems using MAEN, and in the meantime

the gap distance and compliance current were still relatively large to ensure sufficiently long and thick filament(s) for clear observation. Once the SEM observation is replaced by reading and writing periphery, both the gap distance and compliance current can be further reduced, and these will also contribute to the reduction of the solution time and therefore further enhancing the computational efficiency.

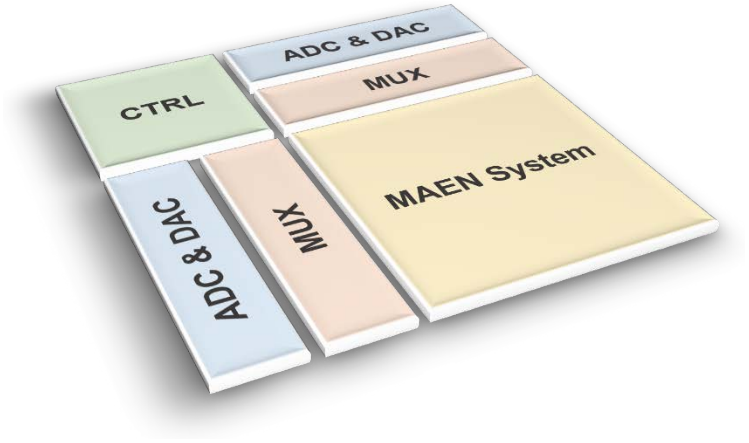

Supplementary Figure 30 | Schematic diagram of circuit platform including writing and reading periphery for MAEN. The module "CTRL" is the controller which sends control signals to decide corresponding operations, such as the terminals to be addressed and the voltage bias to be applied. The multiplexer "MUX" receives control signal from "CTRL" and selects the voltage bias that corresponds to the terminals. The signal converter "ADC & DAC" is used to input and read the analog electrical signal.

Supplementary Note 1:

Discussion on the energy consumption of MAEN system

In this work, relatively large gap distance (hundreds of nanometers) and high compliance current (hundreds of nanoamps to several microamps) were adopted to ensure obvious filament morphology under SEM observations. A rough estimation by directly multiplying the compliance current, applied voltage and forming time leads to relatively high energy consumption from micro to milli Joule. However, it is worth mentioning that during the forming process, the current is always at a low level (from femtoamp to picoamp) before the conductive filament establishes a connection between the terminals, and only reaches the

compliance current after filament formation. Therefore, the actual energy consumption should be calculated by the time integral and should be much lower than the estimated value above, namely,

$$E = \int V \cdot I \cdot \mathrm{d}t \tag{S2}$$

In the future, a dedicated circuit platform including reading and writing periphery can be developed to probe the MAEN result. Once the SEM observation is replaced by reading and writing periphery, both the gap distance (d) and compliance current (I) can be further reduced, and this will also significantly reduce the applied voltage (V) and switching timescale (t). The reduction in compliance current (I), applied voltage (V) and switching time (t) will significantly reduce the power consumption of the MAEN device. Furthermore, the device fabrication processes can also be optimized to further reduce the power consumption. For example, dielectric materials with higher ion mobility can be used to effectively promote Ag movement, which therefore is able to further reduce the applied voltage (V) and switching time (t). The above optimizations in gap distance, compliance current and ion transport properties etc. are expected to be capable of dramatically reducing the power consumption of the MAEN devices.

Supplementary References

S1. Krishnan, K., Tsuruoka, T., Mannequin, C. & Aono, M. Mechanism for Conducting Filament Growth in Self-Assembled Polymer Thin Films for Redox-Based Atomic Switches. *Adv Mater* **28**, 640-648 (2016).

S2. Wang, Z. *et al.* Memristors with diffusive dynamics as synaptic emulators for neuromorphic computing. *Nat Mater.* **16**, 101-108 (2017).

S3. Guan, X., Yu, S. & Wong, H. S. P. On the Switching Parameter Variation of Metal-Oxide RRAM — Part I: Physical Modeling and Simulation Methodology. *IEEE Transactions on Electron Devices* **59**, 1172-1182 (2012).

S4. Gaba, S., Sheridan, P., Zhou, J., Choi, S. & Lu, W. Stochastic memristive devices for computing and neuromorphic applications. *Nanoscale* **5**, 5872-5878 (2013).

S5. Hsiung, C.-P. et al. Formation and instability of silver nanofilament in Ag-based programmable metallization cells. ACS Nano 4, 5414-5420 (2010).
